# The Migration Pattern of a Cementless Hydroxyapatite-Coated Titanium Stem under Immediate Full Weight-Bearing—A Randomized Controlled Trial Using Model-Based RSA

**DOI:** 10.3390/jcm9072077

**Published:** 2020-07-02

**Authors:** Tobias Reiner, Robert Sonntag, Jan Philippe Kretzer, Michael Clarius, Eike Jakubowitz, Stefan Weiss, Volker Ewerbeck, Christian Merle, Babak Moradi, Stefan Kinkel, Tobias Gotterbarm, Sébastien Hagmann

**Affiliations:** 1 Center for Orthopedics, Trauma Surgery and Spinal Cord Injury, Heidelberg University Hospital, Schlierbacher Landstraße 200a, 69118 Heidelberg, Germany; volker.ewerbeck@med.uni-heidelberg.de (V.E.); Christian.Merle@med.uni-heidelberg.de (C.M.); babak.moradi@med.uni-heidelberg.de (B.M.); 2 Laboratory of Biomechanics and Implant Research, Center for Orthopedics, Trauma Surgery and Spinal Cord Injury, Heidelberg University Hospital, Schlierbacher Landstr. 200a, 69118 Heidelberg, Germany; robert.sonntag@med.uni-heidelberg.de (R.S.); philippe.kretzer@med.uni-heidelberg.de (J.P.K.); 3 Department for Orthopedics and Traumatology, Vulpius Klinik GmbH, Vulpiusstrasse 29, 74906 Bad Rappenau, Germany; Michael.clarius@vulpiusklinik.de; 4 Laboratory for Biomechanics and Biomaterials, Department of Orthopaedic Surgery, Hannover Medical School, Anna-von-Borries-Str. 1-7, 30625 Hannover, Germany; Jakubowitz.Eike@mh-hannover.de; 5 ARCUS Clinics Pforzheim, Rastatter Str. 17-19, 75179 Pforzheim, Germany; weiss@sportklinik.de (S.W.); kinkel@sportklinik.de (S.K.); 6 Department for Orthopedics and Traumatology, Kepler University Hospital GmbH, Johannes Kepler University Linz, Krankenhausstrasse 9, 4020 Linz and Altenberger Strasse 69, 4040 Linz, Austria; Tobias.Gotterbarm@kepleruniklinikum.at

**Keywords:** cementless total hip arthroplasty, hydroxyapatite-coated femoral stem, radiostereometric analysis, RSA, migration, implant stability, SL-PLUS MIA

## Abstract

(1) Background: High primary stability is important for the long-term survival of cementless femoral stems in total hip arthroplasty (THA). The objective of this study was to investigate the migration pattern of a hydroxyapatite-coated cementless hip stem developed for minimally invasive surgery using model-based radiostereometric analysis (RSA). (2) Methods: In this randomized controlled trial, 44 patients with an indication for cementless primary THA were randomly allocated to receive either the SL-PLUS MIA stem, developed for minimally invasive surgery, or the SL-PLUS stem (Smith & Nephew Orthopaedics, Baar, Switzerland) which served as a control group. Unlimited weight-bearing was permitted postoperatively in both groups. Model-based RSA was performed after six weeks and after 3, 6, 12 and 24 months postoperatively. (3) Results: Mean total stem subsidence at two-year follow-up was 0.40 mm (SD 0.66 mm) in the SL-PLUS group and 1.08 mm (SD 0.93 mm) in the SL-PLUS MIA group (*p* = 0.030). Stem subsidence occurred during the first six weeks after surgery, indicating initial settling of the stem under full weight-bearing. Both stem designs showed good osseointegration and high secondary stability with no further migration after initial settling. (4) Conclusions: Settling of a cementless straight femoral stem occurs during the first six weeks after surgery under full weight-bearing. Although initial stem migration was higher in the SL-PLUS MIA group, it had no influence on secondary stability. All implants showed good osseointegration and high secondary stability with no signs of implant loosening during this two-year follow-up period.

## 1. Introduction

**Figure 1:**
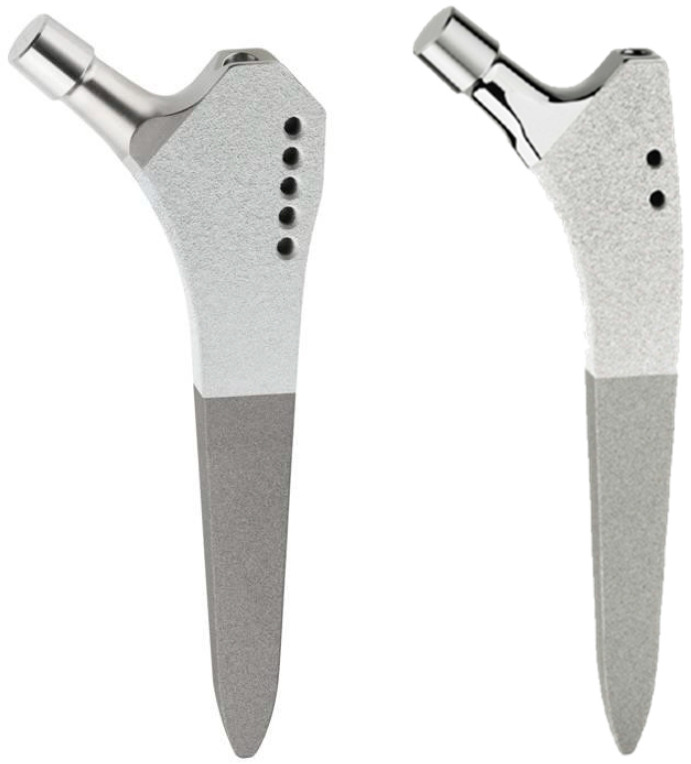
The SL-PLUS stem on the left side and the SL-PLUS MIA stem on the right side. Both stem designs are based on the Zweymüller concept with a proximal hydroxyapatite coating in order to facilitate bone ingrowth. For the SL-PLUS MIA stem the metaphyseal geometry was modified with a less prominent lateral shoulder in order to facilitate a bone preserving implantation.

Cementless total hip arthroplasty (THA) provides excellent long-term results and cementless stems have become the most frequently used implants in primary THA today [1]. Registry data have shown that aseptic loosening remains the most common reason for revision surgery in THA [1]. The initial mechanical stability of the femoral component is an important factor for the long-term survival of the implant. High primary stability allows for early postoperative mobilization under full weight-bearing and is crucial for osseointegration by reducing micromovements along the implant–bone interface [2]. Studies using radiostereometric analysis (RSA) have demonstrated that an increased postoperative stem migration within the first two years after surgery can be a predictor of early implant failure [3,4,5,6]. In addition, torsional instability of cementless femoral components can be associated with unsatisfying functional results and persisting thigh pain [7,8,9]. Due to its high accuracy and the possibility of measuring the three-dimensional position of an object, RSA has been widely accepted as a gold-standard technique for assessing micromotions of orthopedic implants in vivo [10,11]. Besides factors such as surgical technique or bone quality, stem geometry has an important impact on the primary stability of the implant. The cementless hydroxyapatite-coated SL-PLUS femoral hip system (Smith & Nephew Orthopaedics AG, Baar, Switzerland) is based on the dual-tapered, rectangular cross-sectioned concept of the Zweymüller stem. Since its introduction in 1994, it has shown excellent clinical results with a reported cumulative survivorship of 96% at 20 years [12,13]. The SL-PLUS MIA stem was introduced in 2005 to meet the requirements of less invasive surgery. It has a modified metaphyseal stem geometry, which is optimized for soft-tissue preserving approaches and is intended to facilitate implantation with reduced lateral bone resection at the greater trochanter (Figure 1). However, little clinical data for this femoral prosthesis is available.

Therefore, the aim of this prospective randomized study was to assess the migration pattern of the SL-PLUS MIA stem using model-based RSA in order to investigate the influence of the design changes on the primary stability of the implant in comparison with the clinically well-documented SL-PLUS stem, which served as a control group.

## 2. Methods

Forty-four patients, who were allocated for primary THA at our institution between April 2010 and December 2012, were enrolled in this prospective randomized controlled trial to receive one of two types of cementless femoral stems. Sample size calculation was performed using the power analysis software G*Power (Version 3.1.9.3 for Mac) [14]. Given an estimated clinically important difference in stem migration of 0.6 mm between the groups and a standard deviation of 0.6 mm, power analysis indicated that 17 patients would be required in each group in order to achieve a power of 80% in a two-sided t-test with a significance level of 0.05 [15,16]. To compensate for possible dropouts and loss to follow-up, 22 patients were recruited for each group.

The inclusion criteria were patients 35–75 years of age with primary or secondary osteoarthritis of the hip who required cementless THA with a standard offset stem. Exclusion criteria included patients with a body mass index higher than 35 kg/m^2^, rheumatoid arthritis, ongoing corticosteroid or osteoporosis therapy, hereditary skeletal diseases or history of corrective osteotomy of the proximal femur. The study was approved by the local ethics committee (No. S-217/2007) and the Federal Office for Radiation Protection (No. Z 5-2246/2-2007-063) before the inclusion of the first patient. The study was performed in accordance with the Helsinki Declaration and written informed consent was obtained from every patient before study inclusion.

### 2.1. Implants and Surgical Technique

Patients were randomized into one of the two treatment groups using a computer-generated randomization list (nQuery Advisor 5.0, Statistical Solutions Ltd., Ireland). In each group, 22 patients received either the SL-PLUS Standard stem or the SL-PLUS MIA Standard stem (Smith & Nephew Orthopaedics AG, Baar, Switzerland). Both of these cementless femoral components are made of grit-blasted titanium alloy (Ti6Al4Va) with proximal hydroxyapatite coating and have a straight dual-tapered geometry with a rectangular cross-section. The proximal surface of the stem is coated with a 0.3 mm open-pore titanium plasma layer and a 0.05 mm hydroxyapatite (HA) layer with a mean surface roughness Ra of approximately 20–30 µm, in order to facilitate bone ingrowth at the metaphyseal region. The SL-PLUS MIA stem is characterized by a modified metaphyseal stem geometry with a less prominent lateral shoulder as compared with the SL-PLUS Standard stem, which should facilitate a soft-tissue-preserving implantation with reduced bone resection at the lateral trochanter (Figure 1). All patients received a 32 mm diameter ceramic femoral head (BIOLOXforte, CeramTec GmbH, Plochingen, Germany) with the exception of two patients, who received a cobalt-chromium-alloy metal head due to a large femoral offset. On the acetabular side, a cementless press-fit titanium cup (Allofit, Zimmer Biomet, Warsaw, IN, USA) was used in combination with a crosslinked polyethylene insert (Durasul, Zimmer Biomet, Warsaw, IN, USA) in all patients. Two experienced senior surgeons (T.G. and S.W.) performed all surgical procedures according to the manufacturer’s instructions using an anterolateral modified Baur approach. Femoral reaming was performed in a standardized manner with the use of a pneumatic broaching system (Woodpecker, Integrated Medical Technologies USA, LLC, Lino Lakes, MN, USA). Intraoperatively, 5–10 radio-opaque tantalum markers of 1.0 mm diameter were inserted into the cancellous bone of the greater and lesser trochanter using the Halifax Bead Inserter (Halifax Biomedical Inc., Mabou, NS, Canada). Full weight-bearing was allowed immediately after surgery.

### 2.2. Clinical and Radiological Evaluation

The primary outcome measure of the study was the comparison of primary and secondary stability of the femoral component between the study and the control group by measuring stem migration using the model-based RSA technique. Unipolar stereo images were obtained by two synchronized roentgen tubes that were set with an angle of 40 degrees between the crossing X-ray beams. A carbon filter calibration box (Medis Specials, Leiden, The Netherlands) was positioned underneath the patient’s joint of interest with two digital film cassettes placed in the lower plane of the box. The exposure settings were kept constant at 90 kV and 12.5 mAs for all images. Image analysis was performed using model-based RSA (v. 3.3, Medis Specials, Leiden, The Netherlands). According to the RSA guidelines, the upper limit for the mean error of rigid body fitting was kept at 0.35 mm and the upper limit for the condition number was kept at 150 in order to guarantee adequate stability and distribution of the markers [17]. At each follow-up, linear migration of the stem with respect to the baseline measurement was assessed in terms of rotation and translation along all three axes. RSA measurements were performed within the first week after surgery (baseline measurement) and 6 weeks, 3 months, 6 months, 12 months and 24 months postoperatively. In order to determine the precision level of the RSA system, double examinations were performed at the 6-month follow-up visit in a total of 28 patients. Following current recommendations, the patients were repositioned between the examinations and the mean and the standard deviation (SD) of the differences between the two measurements for all patients was calculated [17,18]. Precision was calculated using the formula:

$$P=2.048\times SD=2.048\times \sqrt{\frac{\sum_{i=1}^{n}{\left(x_{i}\right)}^{2}}{n}}$$

where P represents precision, x is the difference between the double examinations, and 2.048 represents the critical value in a 95% t-distribution for a sample size of n = 28 [19].

In addition, standard pelvis anteroposterior and lateral radiographs of the hip were evaluated at a two-year follow-up with regard to radiological signs of implant loosening such as progressive radiolucent lines, osteolysis and/or visible implant migration. Radiolucent lines were considered to be present when they were >1.0 mm and if they occupied more than 50% of the interface in each Gruen zone. Periprosthetic osteolysis was defined as a lucent zone absent of trabecular bone, which was not visible on the immediate postoperative radiograph [20]. Complications were reported at each follow-up visit and the clinical outcome was assessed using the modified Harris Hip Score (HHS). The HHS ranges from 0 (worst) to 100 (best) and considers information on pain, function and range of motion [21]. It was calculated preoperatively and 3, 12 and 24 months after surgery.

### 2.3. Statistical Analysis

Statistical analysis was performed using the software SPSS^®^ for Windows^®^ (version 25.0; SPSS IBM Corp., Chicago, IL, USA) and Graphpad Prism^®^ (version 6.0, Graphpad Software, San Diego, CA, USA). Data were evaluated descriptively as the arithmetic mean, SD, minimum and maximum and 95% confidence intervals. The Shapiro–Wilk test demonstrated normal distribution. In order to compare demographic data and differences in stem migration between the two groups at a given time point, the Student’s t-test for independent samples was used. For comparison of categorical variables between groups, the chi-square test was used. Analysis of variance for repeated measures (ANOVA) with Bonferroni-correction for multiple comparisons was performed to compare differences in stem migration over time. All tests were two-sided and a *p*-value < 0.05 was considered to be statistically significant.

## 3. Results

**Figure 2:**
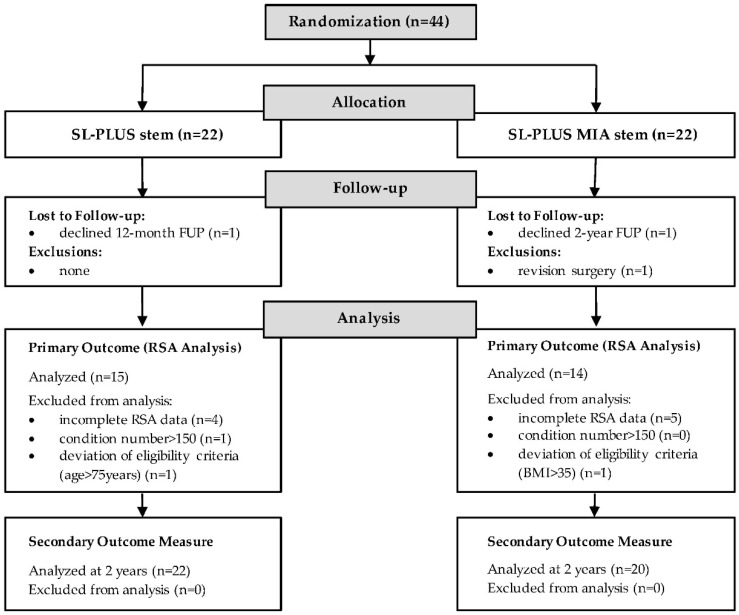
Flowchart demonstrating the randomization and follow-up of patients.

No statistically significant difference was observed between the two groups in terms of baseline demographics or mean preoperative HHS (Table 1). Two patients were lost to follow-up, because they refused the one- and the two-year follow-up visit, respectively. One patient was revised due to aseptic cup loosening at an external hospital. A total of nine patients with incomplete RSA data due to insufficient marker detection and one patient with a condition number >150 had to be excluded for RSA-analysis. In addition, two patients with a deviation of eligibility criteria were excluded, leaving 14 patients in the study group and 15 patients in the control group for RSA-analysis at the two-year follow-up. Randomization and follow-up of patients are summarized in Figure 2.

Two complications were reported. A periprosthetic fracture of the trochanteric tip was observed in the control group at a three-month follow-up in a 57-year-old male patient, which did not require further surgery. One patient treated in the study group reported numbness in the right thigh postoperatively. This was most likely associated with pressure damage of the lateral femoral cutaneous nerve and persisted throughout the follow-up period up to the two-year visit.

**Figure 3:**
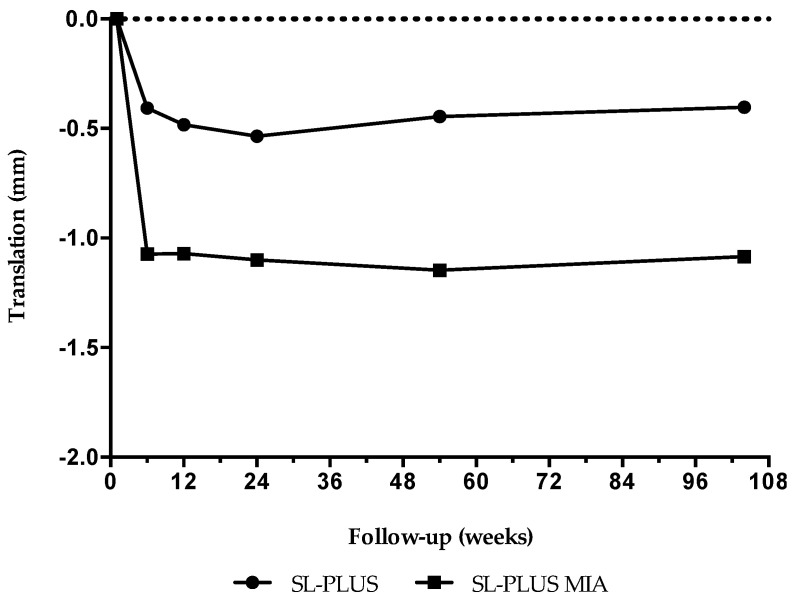
Mean values of stem subsidence (translation along the stem axis) for both femoral components at each follow-up interval. After initial settling within the first 6 weeks no further significant subsidence was noticed for both stem designs.

Table 2 shows the results of double-examination measurements at the six-month follow-up. Mean stem migration along the stem axis was significantly higher in the study group compared with the control group after two years (*p* = 0.030; Table 3). The SL-PLUS MIA stem showed statistically significant subsidence during the first six weeks postoperatively (ANOVA, *p* = 0.014) indicating initial settling of the stem under full weight-bearing with no detectable migration afterwards (Figure 3). Both stem designs demonstrated high secondary stability after initial settling, with good osseointegration and no signs of loosening or persisting migration up to the final follow-up visit two years after implantation. No statistically significant difference in stem rotation and mediolateral translation nor anteroposterior translation was noticed between the two groups and between the follow-up intervals over time. Conventional radiographs at the two-year follow-up showed no signs of radiolucent lines, osteolysis or implant loosening in all patients.

There was a significant postoperative improvement in function in all patients, as demonstrated by the results of the modified HHS. At the two-year follow-up, mean HHS was higher in the SL-PLUS MIA group compared to the SL-PLUS group, although the difference was not statistically significant (*p* = 0.053, Table 4).

## 4. Discussion

The aim of the present study was to assess the stability of the minimally invasive SL-PLUS MIA stem in comparison to the clinically proven SL-PLUS stem using model-based RSA. Both stems showed high secondary stability with excellent osseointegration, although absolute initial subsidence was higher in the SL-PLUS MIA group. No signs of implant loosening or progressive migration after initial settling were noticed during the course of this study.

Excellent long-term clinical results with 20-year survival rates of 96% have been reported in the literature for this stem type [12,13]. Pisecky et al. recently described an overall survival rate of 98% for the Alloclassic stem system after a mean follow-up of 29 years with aseptic stem loosening as the endpoint [22]. However, little clinical data is available to prove the effectiveness and safety of the SL-PLUS MIA stem. Bieger et al. concluded that reducing the shoulder of a Zweymüller stem might negatively influence the rotational stability of the implant [23]. They noticed a less pronounced torque load resistance for the shoulderless prosthesis compared with the original Zweymüller design in their biomechanical study, although the differences between both stems were not statistically significant and overall micromotions were below the critical values [23]. In the context of the high turnover of new prosthesis that is introduced to the market with an expected implant survival time of 20 to 30 years, it is necessary to prove the safety of new stem designs in clinical studies and RSA has shown to be a viable tool to reliably detect future implant failure at an early stage [24].

The mean stem migration measured in the present study was 0.40 mm (SD 0.66 mm) in the SL-PLUS group and 1.08 mm (SD 0.93 mm) in the SL-PLUS MIA group after two years, which mainly occurred within the first six weeks postoperatively. No implant showed continuous excessive migration after initial settling, indicating high secondary stability and consecutive osseointegration in both groups. Increased initial stem migration is a well-known phenomenon of cementless press-fit stems that is not predictive for subsequent loosening. Ström et al. reported a mean two-year subsidence of 1.2 mm (range +0.11 to −6.76 mm) for the clinically well-documented CLS Spotorno stem that took place within the first three months after surgery under early full weight-bearing [25]. Hoornenborg et al. investigated stem migration of the hydroxyapatite-coated SL-PLUS Standard stem in comparison with the uncoated SL-PLUS stem in a recently published RSA study [15]. They did not find a statistically significant difference between both groups and a mean subsidence of 0.46 mm (range −2.17 to 0.05 mm) was reported for the HA-coated SL-PLUS stem after two years, which compares well to our results. This initial migration of straight cementless stems represents implant settling during the early postoperative phase which does not seem to impede secondary stability, as shown by the results of our RSA measurements. In this context, the formerly proposed critical migration rates of 0.5 to 1.0 mm during the first one to two years after surgery, which were considered to be of concern for an increased risk of clinical failure, have to be differentiated [26]. As stated by Campbell et al. it is of importance to evaluate the migration pattern rather than the quantity of stem migration during the first two years when using RSA in order to predict the risk of later implant failure of the straight cementless hip prosthesis [27]. Although there were no signs of continuous stem migration in this cohort, it would be interesting to observe the migration pattern of the SL-PLUS MIA stem into the second decade in order to investigate if the increased initial subsidence would have any negative long-term effects on the clinical outcome, since long-term RSA data of cementless femoral hip stems with a minimum 10-year follow-up are scarce.

The current study could not confirm the findings of others reporting that cementless straight femoral stems tend to rotate into retroversion during the first postoperative year. Ström et al. and Campbell et al. noticed a mean stem rotation into retroversion one year after surgery of 1.90° and 1.79°, respectively [25,27]. Our data did not reveal a statistically significant change of mean stem rotation around the stem axis over time in both groups and no difference was found regarding the rotational stability between the SL-PLUS and the SL-PLUS MIA stem design. The locking mechanism of the Zweymüller stem with a cortical four-point fixation at the proximal diaphysis of the femur due to the rectangular cross-sectional profile could be a possible explanation for the high rotational stability of the stem [28].

The precision levels assessed by double examinations at the six-month follow-up in our cohort were comparable to those reported in the literature by other RSA studies investigating the migration pattern of cementless femoral stems [15,19]. Nysted et al. reported an RSA precision level of 0.21 mm for translation and 1.36 degrees for rotation around the longitudinal axis [19]. Another study by Hoornenborg et al. investigated the same stem design and reported precision values of 0.12 mm for translation and 1.02 degrees for rotation around the longitudinal axis, respectively [15]. However, different approaches in calculating the precision level make a comparison between different RSA studies difficult.

There are limitations to this study that have to be discussed. A high rate of incomplete RSA data was seen in this study, with 10 out of 44 patients not qualifying for final migration analysis. This was mainly due to insufficient marker detection and has to be considered as the main limitation of the present study, as it limits the test power of this study. It underlines the importance of correct RSA marker placement during surgery in order to ensure sufficient marker detection postoperatively, which should be taken into account when planning and conducting future RSA studies. Especially in the region of the lesser trochanter, the sparse amount of cancellous bone can make marker placement difficult although a wide distribution of the tantalum markers around the prosthesis is favorable in order to increase the methodological accuracy and precision of the RSA.

In conclusion, our study demonstrated that the initial subsidence of a hydroxyapatite-coated double-tapered femoral stem occurs during the first six weeks after surgery under full weight-bearing. Initial stem migration was higher in the SL-PLUS MIA group but had no influence on secondary stability. All implants showed good osseointegration and high secondary stability with no signs of implant loosening during this two-year follow-up period. A high rate of insufficient marker detection was seen in this RSA study, which should be taken into account when planning and conducting future RSA studies.

## Figures and Tables

**Table 1:** Demographic data of the study group (SL-PLUS MIA, *n* = 22) and the control group (SL-PLUS, *n* = 22).

Parameter	SL-PLUS MIA (*n* = 22)	SL-PLUS (*n* = 22)	*p*-Value
Age at surgery ^†^ (years)	60 (39–74)	60 (42–82)	0.988
Female gender (%)	68%	64%	0.750
Operated hip (right/left) (n)	10/12	12/10	0.546
Body height ^†^ (cm)	170 (156–187)	169 (156–194)	0.893
Body weight ^†^ (kg)	84 (58–115)	76 (50–123)	0.142
HHS preoperatively ^†^ (points)	50 (28–70)	49 (20–73)	0.754

^†^ The values are given as the mean, with the range in parentheses; HHS = Harris Hip Score.

**Table 2:** The precision (with mean and SD) of radiostereometric analysis (RSA) measurements as investigated by double examinations in 28 patients at the 6-month follow-up.

	Translation (mm)	Rotation (Degrees)
Transverse axis	0.17 (0.10, SD 0.08)	0.45 (0.33, SD 0.22)
Longitudinal axis	0.33 (0.15, SD 0.16)	1.29 (0.87, SD 0.63)
Sagittal axis	0.62 (0.29, SD 0.30)	0.42 (0.14, SD 0.20)

**Table 3:** Results of RSA measurements showing femoral stem migration for both groups at each follow-up interval.

		SL-PLUS (*n* = 15)		SL-PLUS MIA (*n* = 14)		SL-PLUS vs. SL-PLUS MIA
	Time	Mean (SD)	(95% CI)	Mean (SD)	(95% CI)	*p*-Value
**Translation (mm)**						
Medial(+)/Lateral(−)	**6 weeks**	0.02 (0.28)	−0.13 to 0.17	0.06 (0.26)	−0.09 to 0.20	0.729
	**3 months**	0.01 (0.28)	−0.15 to 0.16	0.04 (0.32)	−0.14 to 0.23	0.743
	**6 months**	−0.02 (0.31)	−0.19 to 0.15	0.04 (0.23)	−0.10 to 0.17	0.569
	**12 months**	−0.01 (0.27)	−0.16 to 0.14	0.02 (0.35)	−0.18 to 0.21	0.821
	**24 months**	−0.02 (0.27)	−0.17 to 0.13	−0.03 (0.25)	−0.17 to 0.12	0.927
Proximal(+)/Distal(−)	**6 weeks**	−0.41 (0.83)	−0.86 to 0.05	−1.07 (0.92)	−1.61 to −0.54	0.050
	**3 months**	−0.48 (0.64)	−0.84 to −0.13	−1.07 (0.90)	−1.59 to −0.55	0.051
	**6 months**	−0.54 (0.67)	−0.90 to −0.17	−1.10 (0.86)	−1.60 to −0.60	0.059
	**12 months**	−0.45 (0.76)	−0.87 to −0.02	−1.15 (0.91)	−1.67 to −0.62	0.032 *
	**24 months**	−0.40 (0.66)	−0.77 to −0.04	−1.08 (0.93)	−1.62 to −0.55	0.030 *
Anterior(+)/Posterior(−)	**6 weeks**	0.03 (0.50)	−0.25 to 0.31	−0.03 (0.32)	−0.22 to 0.15	0.701
	**3 months**	−0.20 (0.52)	−0.49 to 0.09	−0.07 (0.56)	−0.40 to 0.26	0.523
	**6 months**	−0.29 (0.64)	−0.64 to 0.07	−0.22 (0.51)	−0.51 to 0.08	0.745
	**12 months**	−0.01 (0.59)	−0.34 to 0.31	−0.18 (0.73)	−0.60 to 0.24	0.499
	**24 months**	−0.22 (0.55)	−0.53 to 0.08	−0.15 (0.59)	−0.50 to 0.19	0.750
**Rotation (degrees)**						
Extension(+)/Flexion(−)	**6 weeks**	−0.29 (0.67)	−0.66 to 0.09	−0.31 (0.69)	−0.71 to 0.09	0.933
	**3 months**	−0.31 (0.63)	−0.65 to 0.04	−0.34 (0.71)	−0.74 to 0.07	0.903
	**6 months**	−0.09 (0.63)	−0.43 to 0.26	−0.02 (0.82)	−0.50 to 0.45	0.823
	**12 months**	0.08 (0.52)	−0.21 to 0.36	−0.22 (0.90)	−0.74 to 0.30	0.277
	**24 months**	−0.03 (0.67)	−0.34 to 0.41	−0.07 (1.10)	−0.70 to 0.56	0.762
Ante(-)/Retroversion(−)	**6 weeks**	−0.12 (1.95)	−1.19 to 0.96	0.63 (1.45)	−0.21 to 1.46	0.256
	**3 months**	−0.48 (1.76)	−1.46 to 0.49	0.55 (3.14)	−1.26 to 2.36	0.280
	**6 months**	−1.15 (1.64)	−2.06 to −0.25	−0.53 (2.62)	−2.04 to 0.98	0.445
	**12 months**	−0.66 (1.48)	−1.48 to 0.16	−0.06 (3.04)	−1.81 to 1.70	0.499
	**24 months**	−1.29 (1.81)	−2.30 to −0.29	−1.03 (2.81)	−2.65 to 0.59	0.766
Valgus(+)/Varus(−)	**6 weeks**	0.02 (0.19)	−0.09 to 0.13	0.07 (0.33)	−0.12 to 0.26	0.652
	**3 months**	0.07 (0.51)	−0.21 to 0.36	0.04 (0.43)	−0.21 to 0.28	0.840
	**6 months**	0.09 (0.47)	−0.18 to 0.35	0.03 (0.43)	−0.22 to 0.28	0.747
	**12 months**	0.01 (0.41)	−0.22 to 0.24	0.04 (0.65)	−0.33 to 0.42	0.876
	**24 months**	0.04 (0.39)	−0.18 to 0.25	0.02 (0.57)	−0.31 to 0.35	0.918

* indicating statistically significant differences between the study and the control group.

**Table 4:** Summary of the clinical results as measured with the Harris Hip Score.

Follow-Up Interval	SL-PLUS MIA Group (*n* = 22)	SL-PLUS Group (*n* = 22)	*p*-Value
Preoperative	50.1 (11.0)	48.8 (14.8)	0.754
3 months	84.2 (11.7)	81.5 (15.2)	0.534
12 months	93.6 (9.1)	90.3 (12.8)	0.354
24 months	95.1 (6.7)	89.0 (11.9)	0.053

The values are given as the mean, with the standard deviation in parentheses.
